# Pragmatic, constructive, and reconstructive memory influences on the hindsight bias

**DOI:** 10.3758/s13423-022-02158-1

**Published:** 2022-08-11

**Authors:** Karolin Salmen, Florian K. G. Ermark, Klaus Fiedler

**Affiliations:** Institute of Psychology, Heidelberg University, Hauptstraße 49/51, 69117 Heidelberg, Germany

**Keywords:** Hindsight bias, Knew-it-all-along effect, Constructive memory, Reconstructive memory, Pragmatics, Saying-is-believing, Audience tuning

## Abstract

In hindsight, when the outcome of an uncertain scenario is already known, we typically feel that this outcome was always likely; hindsight judgments of outcome probabilities exceed foresight judgments of the same probabilities without outcome knowledge. We extend prior accounts of hindsight bias with the influence of pragmatic communication inherent in the task and the consolidation of self-generated responses across time. In a novel 3 × 2 within-participants design, with three sequential judgments of outcome probabilities in two scenarios, we replicated the within-participants hindsight bias observed in the classic memory design and the between-participants hindsight bias in a hypothetical design simultaneously. Moreover, we reversed the classic memory design and showed that subjective probabilities also decreased when participants encountered foresight instructions after hindsight instructions, demonstrating that previously induced outcome knowledge did not prevent unbiased judgments. The constructive impact of self-generated and communicated judgments (“saying is believing”) was apparent after a 2-week consolidation period: Not outcome knowledge, but rather the last pragmatic response (either biased or unbiased) determined judgments at the third measurement. These findings highlight the short-term malleability of hindsight influences in response to task pragmatics and has major implications for debiasing.

Hindsight is 20/20: we just knew that Donald Trump would win the U.S. election in 2016, or we always thought that a global pandemic would occur in the foreseeable future. That an outcome appears more likely after it occurred than in foresight is known as hindsight bias (Fischhoff, 1975; for reviews, see Bernstein et al., 2016; Guilbault et al., 2004; Roese & Vohs, 2012). To demonstrate hindsight bias in the laboratory, two predominant research designs are used (e.g., Pohl, 2007; Pohl & Erdfelder, 2016). In the *hypothetical design*, one group of participants provides naïve answers in foresight, while another group answers in hindsight. For instance, participants read about a newly discovered respiratory virus, which may spread globally (Outcome A) or be eradicated (Outcome B). In the hindsight group, participants learn that a pandemic, Outcome A, occurred (*actual outcome*) but are asked to judge how likely Outcomes A and B are as if they did not know the actual outcome. Nevertheless, participants in the hindsight group typically judge the actual outcome as more likely than participants in the foresight group, who did not learn about the actual outcome. In the *memory design*, participants first give answers in foresight. The same participants then learn about the actual outcome. Subsequently, their task is to recall the answers they gave in foresight. Typically, the recalled answers assign a higher probability to the actual outcome than the original answers given in foresight.

Why does hindsight bias occur? The most prominent and advanced models of hindsight bias, *selective activation, reconstruction, and anchoring* (SARA; Pohl et al., 2003) and *reconstruction after feedback with take the best* (RAFT; Hoffrage et al., 2000), agree in the assumption that knowledge of the actual outcome alters the memory representation of the scenario (Blank & Nestler, 2007). When participants make judgments from hindsight, the assumption is that outcome knowledge causes an irreversible change in the memory representation that subsequently precludes judges from forming unbiased judgments. These memory-based accounts offer viable theories of sufficient conditions for hindsight bias. However, the term hindsight bias encompasses a variety of empirical observations on different measures of inevitability, foreseeability, and recollection (e.g., Nestler et al., 2010) that may stem from fundamentally different mechanisms (see, e.g., Blank et al., 2008; Roese & Vohs, 2012).

Until now, the hindsight bias has proven to be robust and to resist various debiasing attempts (for a comprehensive review, see Son et al., 2021). Are people thus, from the second they receive outcome knowledge, incapable of taking a foresight perspective? The present research aims to add to this discussion and extend prior models of the hindsight bias with two aspects: the influence of communicative pragmatics and memory consolidation of task information and self-generated responses across time. We outline how hindsight bias can be explained as a malleable response to pragmatic demands of the task and how the same process can enable unbiased judgments even while having outcome knowledge. However, memory consolidation constrains this assumed flexibility and leads to long-term representations of either biased or unbiased responses.

## Preview of the present research

**Fig. 1:**
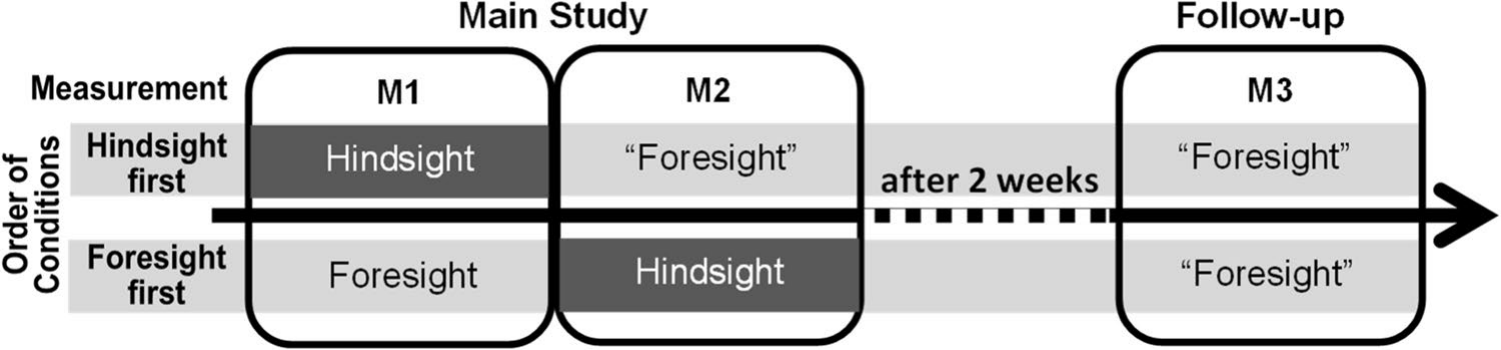
Within-participants design calling for repeated outcome probability judgments of two scenarios across three measurement points (M1–M3). Participants estimate the outcome probabilities for the two scenarios in different sequential order conditions (foresight-first vs. hindsight-first). We denote measurements in which participants receive foresight instructions, but already possess outcome knowledge from a prior measurement, as “foresight”

To illustrate the experimental approach used to demonstrate this, consider the repeated-measures design depicted in Fig. 1. For two different scenarios with two possible outcomes (A vs. B), each participant provides probability estimates at three measurement points (M1–M3), but in a different order of instructions per scenario. In the *hindsight-first* order (upper sequence of Fig. 1), participants first receive hindsight instructions, which include the actual outcome. However, they are told to always respond as if they did not have outcome knowledge. Directly after that, at M2, they encounter the same scenario without the actual outcome (foresight instruction). Of course, they already possess outcome knowledge from the prior trial; we thus call this condition “foresight” (with exclamation marks). The *foresight-first* sequence for the other scenario (lower sequence in Fig. 1) starts with a genuine foresight judgment at M1, followed by a hindsight judgment when participants are informed about the actual outcome at M2. 2 weeks later at M3, participants respond to both scenarios again under foresight instructions. Naturally, this is a “foresight” judgment, as participants possess outcome knowledge from either M1 (hindsight-first) or M2 (foresight-first).

Thus, comparing M1 and M2 judgments in the foresight-first condition allows us to test whether subsequent hindsight judgments reproduce previous foresight judgments. This is a variant of what Pohl (2007) calls a memory design that instructs participants to exclude outcome knowledge from their judgments but does not directly ask for recall of prior judgments. The hindsight-first condition offers a completely novel reversal of this design: Do participants manage to construe typical foresight judgments at M2, even though they already have outcome information from preceding hindsight judgments at M1?

Within the same design, a comparison of M1 judgments in the hindsight-first and foresight-first conditions resembles what Pohl (2007) calls a hypothetical design. Here, hindsight bias means that participants who receive a scenario with the actual outcome at M1 (hindsight-first) judge this outcome as more likely than participants who receive the same scenario with foresight instructions at M1 (foresight-first).

With the final M3 judgment, we test participants’ long-term problem representation, after a delay of 2 weeks. If the crucial determinant is outcome knowledge, which participants either received at M1 or M2, both M3 judgments should resemble a typical hindsight judgment. If, however, M3 judgments are sensitive to memory for the participants’ own previous judgments and to pragmatic influences of previously communicated probabilities, the influence of their own prior behavior may dominate the impact of existing outcome knowledge.

Let us elaborate on the pattern of empirical findings that can be expected on theoretical grounds. The mechanistic accounts cited at the outset agree in predicting that in the crucial hindsight-first condition, participants should exhibit hindsight bias in M1 and M2. They could hardly explain why “foresight” judgments at M2, after genuine outcome knowledge is induced at M1, should resemble naïve foresight judgments given in the absence of outcome knowledge. However, exactly such a pattern of malleable and reversible hindsight effects, regardless of prior outcome knowledge can be derived from a pragmatic theory perspective, if we assume that participants can frame their judgments according to given linguistic-pragmatic constraints.

Consistent with principles of pragmatic communication (Grice, 1975; Schwarz, 1996), participants may not be bound to generating factual judgments that reflect the entirety of all relevant information about the scenarios. Rather, they may be capable of construing the same judgment in different ways, depending on pragmatic task constraints, such as communication partners’ attitudes and an understanding of the intentions guiding their communication (e.g., Higgins & McCann, 1984).

Specifically, regarding the repeated-measures design in Fig. 1, we assume that prior to the 2-week memory consolidation between M2 and M3, probability judgments are quite malleable, allowing the participants to construe the outcome probabilities of the same scenarios in flexible ways. It is likely that when they receive the typical hindsight instruction, participants believe it to be pragmatically appropriate to construe their judgments in line with the outcome knowledge, which their communication partner, the experimenter, features so prominently. In such a task setting, even an explicit instruction to ignore the outcome may only reinforce, rather than reduce, the focus to the outcome as a relevant cue for mental construal. Meanwhile, at M2 in the hindsight-first order, the experimenter asks participants for the same judgment again, while the outcome notably disappears from the task. This presumably communicates to participants that giving a judgment that differs from their prior answer and excludes outcome knowledge is now appropriate.

When judges indeed respond to pragmatic task constraints, they should judge the actual outcome as more likely under hindsight instructions (with actual outcome) than under foresight instructions (without actual outcome), regardless of the order of instructions. Thus, from this perspective, we not only expect to replicate the typical finding of a memory design (i.e., increasing probability judgments of the actual outcome from foresight at M1 to hindsight at M2) but also the seemingly paradoxical reversal (decreasing probability judgments from hindsight at M1 to “foresight” at M2), despite the available outcome knowledge.

- *Hypothesis 1:* Likelihood judgments of the actual outcome are higher under hindsight instructions than under foresight (or “foresight”) instructions, independent of the sequential ordering of both judgment conditions.

### Constraints on constraints

Yet the malleability of probability judgment construal is not unlimited. Referring to Hastie and Park’s (1986) distinction of memory-based and on-line judgments, we expect judgments to be malleable while the online construal process is still ongoing rather than complete. The time range of the malleable phase can be expected to cover M1 and M2, during which participants receive the same scenario twice in direct succession, without any extraneous mental activities and consolidation period in between. However, once a 2-week delay between M2 and M3 provides ample time for consolidation, we assume that a strong preformed judgment in memory becomes inevitable. Accordingly, we predict a distinct shift from malleable online construal of judgments (during M1 and M2) to preformed memory-based judgments at M3, with preceding M2 judgments being the strongest predictor of the final M3 judgments.

We propose that repeated responses in a hindsight paradigm are subject to a pronounced generation effect, that is, a selective memory bias in subsequent judgments toward self-generated information from preceding judgments (Bertsch et al., 2007; Slamecka & Graf, 1978). A canonical exemplar of such a self-generation effect in cooperative communication is the saying-is-believing effect (Hardin & Higgins, 1996; Higgins, 1992, 1999; Higgins & Rholes, 1978). It is typically observed within the audience-tuning paradigm, where participants describe a person to another person. When participants tune this description toward the attitude of the audience, this also shapes their own memory representation and attitude toward the described person. For this effect to occur, participants need not initially believe in their answer or hold it as the single best estimate (Hardin & Higgins, 1996; Higgins, 1992, 1999). People are not only accountable for what they communicate to others (Tetlock, 1983); the well-known memory advantage of self-generated information also implies that one’s own previously communicated self-generated judgments may exert a similarly strong, or even stronger, impact on one’s subsequent knowledge than experimenter-provided outcome information. Thus, the high- or low-probability judgment induced by the pragmatic prompts at M2 should determine the final M3 judgment.

- *Hypothesis 2:* After 2 weeks, likelihood judgments of the true outcome made under “foresight” instructions (M3) resemble the last judgments that participants provided (M2).

Note that the predicted pattern diverges in distinct ways from prior mechanistic accounts, which converge in predicting that participants cannot stop using available outcome knowledge. The pragmatic perspective does not require a distinction between “legitimate” and “forbidden” information (e.g., Fischhoff, 1975; Pohl & Hell, 1996) or a rationalist assumption that the intrusion of (forbidden) outcome knowledge is in line with Bayesian updating processes (Dietvorst & Simonsohn, 2019). To set our pragmatic account apart from the assumption that hindsight effects constitute deliberate attempts to overcome inaccuracy and bias, we included a measure of participants’ metacognitive insights in judgment biases in the following research.

## Method

### Materials

We adopted the four stimulus scenarios from Slovic and Fischhoff (1977). Each scenario involved two possible outcomes (A and B). For instance, in one scenario researchers successfully influenced a hurricane with a chemical, with the outcomes “the strength of the hurricane increased” (A) and “the strength of the hurricane decreased” (B). Under foresight instructions, participants read the vignette and then immediately judged the probability of both outcomes. In the hindsight instructions, the sentence “The actual outcome was (A/B)” was appended to the vignette. The actual outcome was A for two scenarios and B for the two other scenarios. The computer dialogue forced participants to enter two percentages (one for each outcome), which had to sum up to 100%. Within this paper, we always report the judged probability of the actual outcome as the relevant judgment, meaning that an increase in judgments under hindsight instructions compared with foresight instructions shows hindsight bias. The scenario and actual outcome for hindsight instructions remained visible during the judgments. The entire experiment was administered as an online questionnaire using SoSci Survey (Leiner, 2020). Instruction provision and data collection were fully automated, warranting complete anonymity and eliminating all experimenter influences.

### Design and procedure

The repeated-measures design is summarized in Fig. 1. Every participant received two scenarios, for which three measurement points (M1, M2, M3) were collected: M1 and M2 in the main study and M3 2 weeks later in the follow-up. For one scenario, participants received hindsight instructions at M1 and then foresight instructions at M2 (*hindsight-first*; upper sequence in Fig. 1). For the other scenario, they started with foresight instructions at M1, followed by hindsight instructions at M2 (order called *foresight-first*; lower sequence in Fig. 1). The allocation of scenarios to the order of instructions was counterbalanced, as were the scenario pairs allocated to a given participant. Whether the foresight-first or hindsight-first condition appeared first was also counterbalanced. In summary, we employ a 2 × 3 within-participants design with the factors order of instructions (foresight-first, hindsight-first) and measurement (M1, M2, M3).

In the main study (collection of M1 and M2), participants first received detailed instructions. We explicitly informed all participants about the task and the typical differences between probability judgments made in foresight and hindsight (the bias regularly occurs even if participants are aware of it, see, e.g., Pohl & Hell, 1996). They were also informed that the study would continue 2 weeks later. They then responded twice to each of two scenarios. Judgment prompts for each scenario appeared on a new page, asking participants to provide two types of ratings: the probability of each outcome and a metacognitive self-report measure in which we asked participants to indicate on a 7-point scale which of the typical responses their judgment resembled more (from 1 = *foresight* to 7 = *hindsight*). Please note that the initial instructions about the hindsight bias enabled participants to base this comparison on a common standard. When participants received the same scenario for a second time (M2), they started with the following instructions: “Now, reread the scenario. If you received outcome information on the previous page, try not to use it for your judgments.” Upon completion of two judgments for both scenarios, participants provided demographic data. The duration of the first session was about 7 minutes (3 to 12).

Roughly 2 weeks later, participants were invited again to the follow-up (collection of M3). Now all participants received all four scenarios under foresight instructions, with the following additional instruction: “You already know two of these scenarios from the first survey on this topic. Please respond as you did in the foresight condition.” Again, each scenario appeared on a new page, in random order. For each of the scenarios, we asked participants to indicate their metamemory experience. Three response options were provided: “*This scenario is new to me*,” “*I know this scenario*,” and “*I remember my prior responses to this scenario exactly*.” This question includes two relevant aspects: whether an item is recognized (yes/no) and, if so, whether participants merely know that they have seen the scenario or have a recollective experience, remembering the precise episode and which judgments they had given in the main study. Note that the latter distinction is aimed to discriminate between semantic and episodic memory (Tulving, 1972), operationalized as “remember” versus “know” (Rajaram, 1993). The total follow-up lasted about 4 (3–7) minutes.

### Participants

The main study included 90 participants (68 female, *M*_age_ = 24.74 years, range: 20–55 years); 64 of them remained in the follow-up (48 female, *M*_age_ = 25.09 years, range: 20–55 years). The target sample size was determined by a simulation-based power analysis documented in the Appendix. We recruited participants from social psychology courses for bachelor students at Heidelberg University. Participation was unpaid. Participants could choose, both at the start and at the end of the session, to preclude us from analyzing their data (three excluded for this reason). Five further participants were excluded due to irregularities (repeated participation, less than a day from the main study to follow-up). Incomplete attempts were also deleted (main study: *n* = 12, follow-up: *n* = 1). On average, the delay between main study and follow-up was 13.5 days (9–21). Participants provided informed consent before each study part and were debriefed at the end. We completed data collection before data analysis commenced.

### Statistical analyses

For our analyses, we used R (Version 3.6.1; R Core Team, 2019), as well as the packages lme4 (Bates et al., 2015), lmerTest (Kuznetsova et al., 2017), sjPlot (Lüdecke, 2019), simr (Green & MacLeod, 2016), and ggplot2 (Wickham, 2016). We used mixed-effects modeling to account for our longitudinal repeated-measures design. To aid readability, we provide the results in the more common analysis of variance (ANOVA) format within the main text. The interested reader finds the full mixed-effects model analysis in the Appendix.

## Results

### Probability judgments—Main study and follow-up

**Fig. 2:**
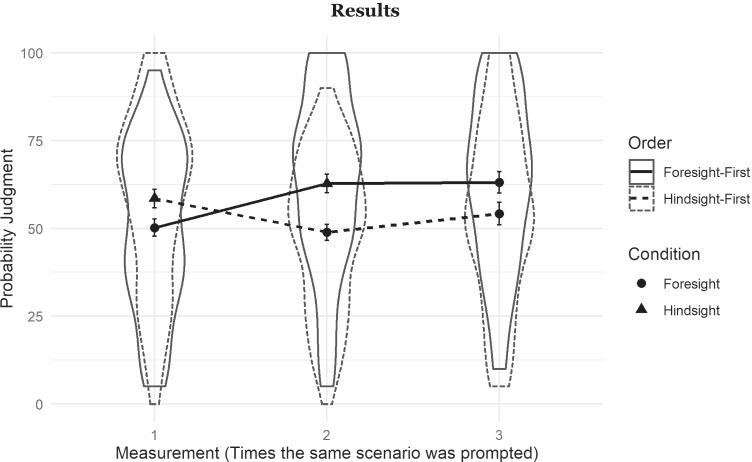
Violin plot with corresponding line graph depicting the means, standard errors and distributions of outcome probability judgments as a function of measurement (M1, M2, M3), condition (circles vs. triangles), and ordering of foresight and hindsight condition (solid vs. dashed lines)

Figure 2 displays the judged probability of the actual outcome across conditions and measurements. We replicated the classic between-participant hindsight bias in the hypothetical design. At M1, probability judgments under the hindsight instruction (*M* = 58.85%) were significantly higher than under the foresight instruction, with no outcome knowledge (*M* = 49.83%), *b* = 8.60, *t*(88.37) = 2.25, *p* = .01, *d* = .36. The classic within-participant finding in the memory design was also replicated: After participants had provided judgments under foresight instructions at M1 (*M* = 49.83%), they judged the actual outcome as more likely under hindsight instructions at M2 (*M* = 62.79%), *b* = 12.60, *t*(89) = 5.92, *p* < .001, *d* = .52.

#### Hypothesis 1

In the critical hindsight-first order, the mean probability estimate for the actual outcome decreased from 58.85% with hindsight instructions at M1 to 49.22% with foresight instructions at M2. This decline from hindsight to “foresight” constitutes a well-tuned downward adjustment, despite formerly induced outcome knowledge, matching the strength of the classic between- and within-participants hindsight bias.

These results were reflected in a two-way repeated-measures ANOVA with the factors instruction (foresight, hindsight) and measurement (M1, M2). There was a significant main effect of instruction: likelihood judgments were generally higher under hindsight instructions (*M* = 60.66) than under foresight instructions (*M* = 49.54), *F*(1, 264.93) = 27.82, *p* < .001. The measurement (M1, M2) did not have a significant influence on judged likelihood, *F*(1, 266.19) = 1.18, *p* = .28. There was also no interaction between instruction and measurement, *F*(1, 264.93) = 0.50, *p* = .48. A stable hindsight bias was obtained regardless of whether the foresight condition was followed or preceded by the hindsight condition (see Fig. 2). Put differently, participants provided judgments that resembled genuine foresight even when they possessed outcome knowledge.

#### Hypothesis 2

Two weeks later at M3, likelihood judgments of the true outcome made under foresight instructions mirrored the judgments that participants provided at M2. The mean judged probabilities of the actual outcomes in the foresight-first (*m* = 63.16%) and the hindsight-first order (*M* = 54.22%) were still significantly different, *b* = 8.61, *t*(57.36) = 2.25, *p* = .028, *d* = .34.

Thus, when participants concluded the main study with a foresight judgment (hindsight-first order), there was less hindsight bias after 2 weeks than when the main study concluded with hindsight instructions (foresight-first order). When participants last judged under hindsight instructions before the consolidation period of 2 weeks, they showed a pronounced hindsight bias despite receiving foresight instructions at M3.

### Metacognitive insight—Main study

The responses to the metacognitive insight scale followed a pronounced bimodal distribution. We therefore dichotomized responses (1–3 changed to 0, close to foresight, and 5–7 to 1, close to hindsight) and excluded responses on the median value of the scale (4), as their interpretation is ambiguous. After transformation, 180 responses were coded as “similar to foresight” (0) and 133 responses as “similar to hindsight” (1).

In the foresight-first order, participants indicated less frequently that their judgment was “similar to hindsight” (*M*_Foresight_ = 39%, *M*_Hindsight_ = 36%) than in the hindsight-first order (*M*_Foresight_ = 46%*, M*_Hindsight_ = 49%). This is at odds with participants’ actual judgments. Their responses in the main study depended only on the judgment condition, not on the presentation order. Suppose participants were fully sensitive to the difference in their provided judgments. In that case, the received instruction (hindsight vs. foresight) and not the presentation order should guide their responses. Still, participants seem to think that the outcome influences them in the hindsight-first order, but not the foresight-first order, independent of the trial’s actual condition, thereby contradicting their probability judgments.

### Metamemory—Follow-up

Recognition of the scenarios at the follow-up was almost perfect, with two false alarms in 256 responses. Therefore, we attempted no further analyses of recognition rates. Of the 128 recognized items, participants indicated 92 as known (72%) and 36 as remembered precisely (28%).

**Fig. 3:**
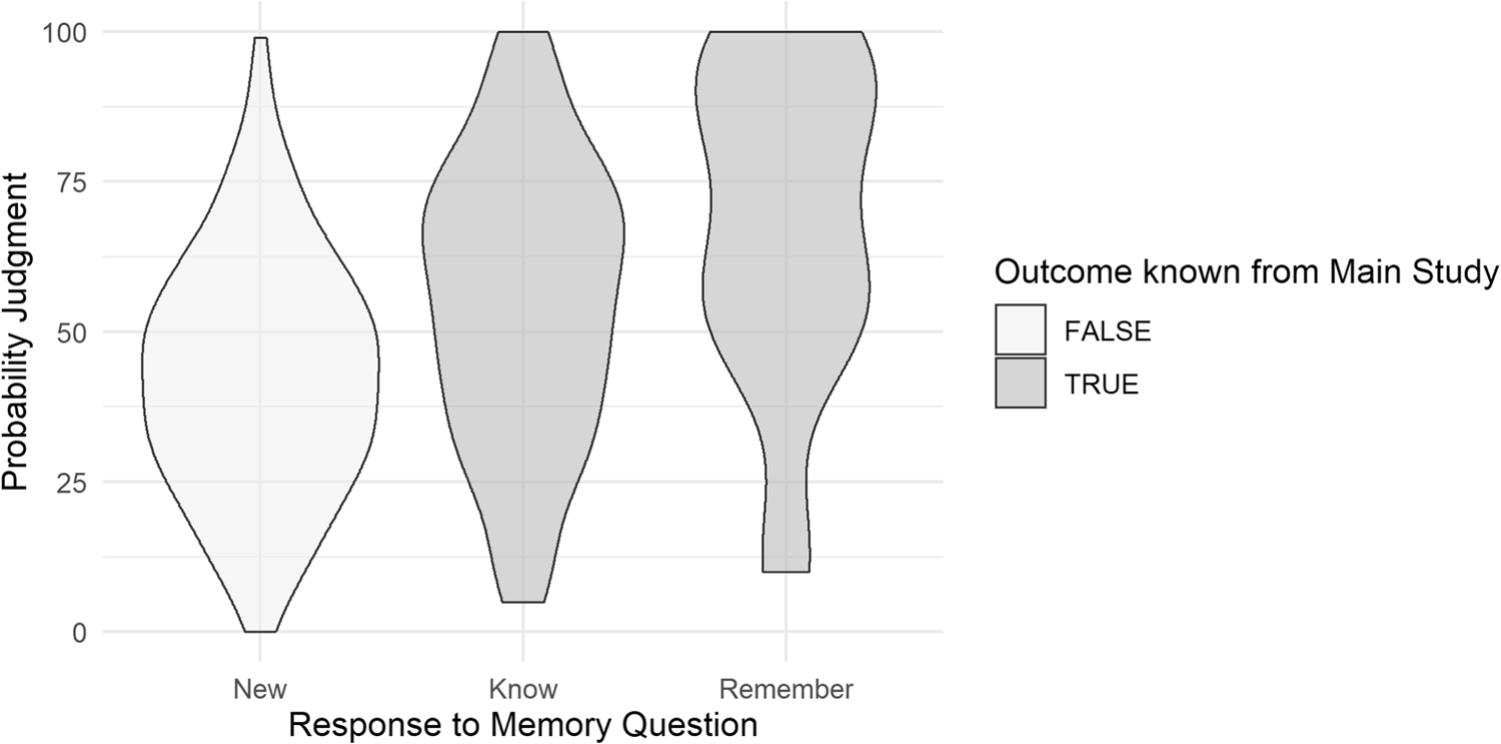
Distribution of probability judgments for the true outcome at the follow-up as a function of outcome knowledge and self-reported metamemory experience

When participants reported that the scenario was new to them, the mean judged probability of the true outcome was 42.96%, 95% CI [35.35, 50.56], *p* < .001. It increased by 11.38% for trials in which participants knew the scenario, 95% CI [5.50, 17.27], *p* < .001, *d* = .50. With 24.61%, this increase was highest when participants reported that they remembered their main study’s responses exactly, 95% CI [16.47, 32.76], *p* < .001, *d* = 1.07. The persistent influence of the outcome information at the follow-up is visible in the descriptive data provided in Fig. 3. Surprisingly, reported recollective memory of preceding judgment scenarios did not lessen the hindsight bias but even strengthened it.

## Discussion

Within our novel experimental design, we find a completely reversible, symmetrical pattern of hindsight effects at M1 and M2, while the judgments made after a 2-week consolidation period at M3 mirror the judgments at M2. Thus, we find a malleable construal effect in response to pragmatic demands while judgments are made on-line: participants succeeded in providing unbiased responses, even if they possessed outcome knowledge and just gave biased responses under hindsight instructions. After consolidation, this malleability is replaced by memory-based judgments consistent with participants last responses. This finding aligns with research on constructive memory (e.g., Loftus, 1975), audience tuning, and the saying-is-believing effect (Hardin & Higgins, 1996; Higgins, 1992, 1999). When no other communicative or self-generated prompts occur, the labile representations formed in cooperative communication become part of a consolidated, stable memory representation.

This distinct pattern of findings is plausible from our theoretical approach, which considers pragmatics and constructive memory, but it can hardly be explained by prior mechanistic accounts of hindsight effects. Interestingly, participants had minimal metacognitive insight into their responses, which does not align with recent claims that the biased responses in the hindsight paradigm reflect a deliberate choice of participants (Dietvorst & Simonsohn, 2019).

We explored the interplay of pragmatics, constructive, and reconstructive memory using predictions for fictional experimental settings as an exemplary task from the literature. Further studies should replicate these findings with other material, such as almanac questions and historical events, while also including foreseeability, inevitability and confidence in judgments (Ackerman et al., 2020) as dependent variables. Additionally, our understanding of cooperative communication as an influence on hindsight bias can be broadened by introducing different linguistic-pragmatic constraints, like the instructions of dialectical bootstrapping (Herzog & Hertwig, 2009). Further research is also needed to determine whether the long-term influence of pragmatic answers within our study shares the typical moderators of saying-is-believing effects, such as audience characteristics and communicative goals (Echterhoff et al., 2005; Echterhoff et al., 2008).

The flexibility of answers given in communication and the long-term constructive influence of these answers on memory oppose a narrow definition of rationalist updating towards more accurate knowledge. However, we argue that both the malleability of answers to fit the communicative context and their long-term influence should be considered adaptive in a world in which accurate predictions, but also social bonds and acceptance by others, determine success. Reacting to communicative-pragmatic constraints and forming a stable shared representation allows us to further our social goals and effectively act together.

Importantly, we also demonstrated how this mechanism can lead to more accurate judgments. Sometimes, for instance after receiving inaccurate outcome information, learning from feedback leads to less accurate judgments. When social sharing prompts someone to exclude inaccurate information directly after receiving it, our findings suggest that they will often succeed. Crucially, we demonstrated that this correction can be temporally stable. If we aim to debunk false claims and enhance prediction accuracy, knowledge of the subtle, low-effort manipulation used in the present research could inspire effective communicative strategies and interventions.

This investigation has demonstrated how communicative malleability in response to communicative-pragmatic constraints, over time, becomes an inextricable part of our memory. Hindsight effects can stem both from the reconstructive nature of memory and the constructive malleability necessary in an uncertain and social world.

## Appendix

### Power analysis

To determine the size of the participant sample, we conducted a power analysis by simulation (see, e.g., Brysbaert & Stevens, 2018), based on Slovic and Fischhoff’s (1977) results with the same materials, assuming a conservative difference between foresight and hindsight (i.e., *p* = .10 compared with *p* = .17 in the original study). The estimated sample sizes required to reach at least .80 power in the planned mixed-effects models was 57 (probability judgments, both main study and follow-up), 68 (metacognitive insight judgments, only main study), and 46 (metamemory judgments, only follow-up). As we expected substantial attrition from the main study to the follow-up, we planned the main study to exceed these estimates.

### Mixed-effect models

We used a linear mixed-effects model to analyze probability judgments. This allowed us to adequately represent the longitudinal repeated-measures design, use all collected data within one model despite of attrition at M3 and comprehensively report the multiple contrasts relevant to our hypotheses. Following the experimental design, we used the predictors order of instructions, measurement, and their interaction. Factors were dummy coded, treating hindsight-first order and M1 as the baseline. We indicate effect sizes using *ICC* for random effects and *d* for mixed-effects models (following Brysbaert & Stevens, 2018; Westfall et al., 2014).

#### Probability judgments

The linear mixed-effects model includes random intercepts for both participants and scenarios, as they are a sample of possible material we seek generalization from (Judd et al., 2017). It was implemented in lmer with the following code:

Judgment ~ Order * Measurement + (1|Participant) + (1| Scenario)

Appendix Table 1 summarizes the results of this model. At M1, probability judgments in hindsight (*m* = 58.85%) were on average 9.02% higher than in the foresight condition (*m* = 49.83%) replicating the classic between-participant finding. Moreover, the same hindsight effect was obtained regardless of whether the foresight condition was followed or preceded by the hindsight condition (see Fig. 2). Given the hindsight-first ordering, the mean outcome probability estimate decreased by 9.63% from 58.85% at M1 to 49.22% at M2. This decline from hindsight to foresight constitutes a well-tuned downward adjustment, despite formerly induced outcome knowledge, matching the strength of the classic between-participants hindsight bias at M1. After 2 weeks, the adjustments made from M1 to M2 persisted at M3 when the last estimate was given in the hindsight condition (foresight-first order). In the hindsight-first order, the difference between M1 (hindsight condition) and M3 does not reach statistical significance anymore.

#### Metacognitive comparison to foresight and hindsight condition

We analyzed dichotomized self-classification data of judgments according to the foresight versus hindsight distinction as a function of scaled probability judgments and judgment conditions in the following logistic regression model: Similar To Hindsight ~ scale(Judgment) + Order * Condition. Due to variance in random effects approaching zero, they led to fit issues and were excluded from the model.

Increasing the probability judgment by one standard deviation makes it 1.84 times more likely that the participant responds “close to hindsight” (see Appendix Table 2). However, after accounting for differences in judgments and order, the judgment condition (hindsight vs. foresight judgment) has no significant influence. Including the judgment condition into the model does not significantly contribute to model fit, χ^2^(2) = 2.07, *n.s*. To quantify this null result further, we computed the Bayes factor for the model comparison (Rouder et al., 2009): once again, the model without condition is preferred, *BF* = 28.93 ±3%.

#### Metamemory self-report

We analyzed the dichotomized responses as a function of scaled probability judgments and judgment conditions in a logistic regression model: Judgment ~ Memory Scale + (1|Scenario). The resulting model is summarized in Appendix Table 2. The metamemory self-report was dummy-coded, with the “new” response as the baseline. We removed participant random effects due to variance close to zero. Scenario random effects remained (τ = 44.94, residual variance σ^2^ = 481.37, *ICC* = 0.09). Overall, the model was fit on 256 observations, with marginal / conditional *R*^2^ = 0.124 / 0.199.

**Table 1:** Results of the linear mixed-effects model of probability judgments

Predictors	Probability judgment			
	Estimates	95% CI	*p*	*d*
Intercept: Measurement 1, Order hindsight-first	58.85	51.27, 66.43	**<.001**	**2.41**
Order: foresight-first	−9.02	−15.10, −2.95	**.004**	**0.37**
Measurement 2	−9.63	−15.70, −3.57	**.002**	**0.40**
Measurement 3	−4.44	−11.18, 2.29	.197	0.18
Order: foresight-first * Measurement 2	22.23	13.65, 30.81	**<.001**	**0.91**
Order: foresight-first * Measurement 3	17.73	8.31, 27.15	**<.001**	**0.73**
Random Effects				
σ^2^	431.13			
τ_00 Participant_	128.18			
τ_00 Scenario_	34.96			
*ICC*	0.27			
*N*_Participant_	90			
*N*_Scenario_	4			
Observations	488			
*R* ^2^	Marginal .051 / Conditional .311			

Single-estimate *p* values are based on the Satterthwaite approximation. Marginal and conditional *R*^2^ statistics rely on Nakagawa and Schielzeth (2013), *d* on Westfall et al. (2014).

**Table 2:** Results of the Logistic Regression predicting Ratings on the Metacognitive Insight Scale

Predictors	Similar to hindsight		
	Odds ratios	95% CI	*p*
Intercept: Order hindsight-first, Hindsight Condition	0.88	0.55, 1.39	.573
scale(Judgment)	1.84	1.43, 2.37	**<.001**
Order foresight-first	0.46	0.24, 0.90	**.024**
Foresight Condition	1.11	0.57, 2.16	.766
Order foresight-first: Foresight Condition	1.48	0.58, 3.79	.414
Observations	313		
Tjur’s *R*^2^	.087		
